# Yeast (1,3)-(1,6)-beta-glucan helps to maintain the body’s defence against pathogens: a double-blind, randomized, placebo-controlled, multicentric study in healthy subjects

**DOI:** 10.1007/s00394-013-0492-z

**Published:** 2013-01-23

**Authors:** Annegret Auinger, Linda Riede, Gordana Bothe, Regina Busch, Joerg Gruenwald

**Affiliations:** analyze & realize ag, Waldseeweg 6, 13467 Berlin, Germany

**Keywords:** Randomized, Placebo-controlled, Double-blind study, Insoluble yeast beta-glucan, Common cold, Immune system

## Abstract

**Purpose:**

The effect of brewers’ yeast (1,3)-(1,6)-beta-d-glucan consumption on the number of common cold episodes in healthy subject was investigated.

**Methods:**

In a placebo-controlled, double-blind, randomized, multicentric clinical trial, 162 healthy participants with recurring infections received 900 mg of either placebo (*n* = 81) or an insoluble yeast (1,3)-(1,6)-beta-d-glucan preparation (*n* = 81) per day over a course of 16 weeks. Subjects were instructed to document each occurring common cold episode in a diary and to rate ten predefined infection symptoms during an infections period, resulting in a symptom score. The subjects were examined by the investigator during the episode visit on the 5th day of each cold episode.

**Results:**

In the per protocol population, supplementation with insoluble yeast (1,3)-(1,6)-beta-glucan reduced the number of symptomatic common cold infections by 25 % as compared to placebo (*p* = 0.041). The mean symptom score was 15 % lower in the beta-glucan as opposed to the placebo group (*p* = 0.125). Beta-glucan significantly reduced sleep difficulties caused by cold episode as compared to placebo (*p* = 0.028). Efficacy of yeast beta-glucan was rated better than the placebo both by physicians (*p* = 0.004) participants (*p* = 0.012).

**Conclusion:**

The present study demonstrated that yeast beta-glucan preparation increased the body’s potential to defend against invading pathogens.

## Introduction

Cumulative evidence obtained from animal models and human studies strongly suggest the immunostimulatory effect of beta-glucans [1]. Beta-glucans are natural polysaccharides consisting of a backbone of (1,3)-beta-glycosidic linked d-glucose subunits, with irregular beta-(1,6)-linked side chains of various length. They are major structural components of the cell walls of brewers’ yeast *Saccharomyces cerevisiae*, fungi and some bacteria. Depending on the source, there are clear differences between beta-glucans in their solubility, molecular mass, tertiary structure, degree of branching, polymer charge and solution conformation, all of which in turn impact their immune modulating effects [2, 3]. Accordingly, beta-glucans having a (1,3)-beta chain with (1,6)-beta branching are more effective than ß-(1,3) branching alone [2].

A properly functioning immune system is crucial to defend against invading pathogens, including common cold viruses. During a cold episode, the virus comes into contact with receptors on epithelial cells of the respiratory tract which in turn triggers a cascade of innate and adaptive immune response mediated by the release of inflammatory cytokines [3]. The common cold is a well-known and, in most cases, harmless infection, typically affecting adults 2–5 times per year but resulting in significant effects on health, well-being and labour productivity [3]. Indeed, each cold episode experienced by working adults leads to an average of 8.7 lost work hours [4].

Very recently, the effect of an insoluble yeast (1,3)-(1,6)-beta-d-glucan on common cold has been demonstrated in healthy subjects [5]. In this study, the incidence of common cold episodes was used as a model system to determine the effect of beta-glucan supplementation on the body’s defence mechanism. The present interventional trial was conducted to substantiate these data.

## Materials and methods

### Study population and investigational product

Two hundred and twenty-four healthy subjects with recurring common colds were included in the study. They had to meet the following inclusion criteria: age ≥ 18–70 years, written consent to participate, and at least three common cold infections within the last 6 months. The main exclusion criteria were as follows: acute or chronic upper airways disease, chronic cough, chronic rhinitis (e.g. allergic rhinitis) or asthma, severe organ or systemic disorders, stomach or intestinal diseases, congenital or acquired immunodeficiency diseases, vaccination against influenza or swine flu within 21 days before the study start, suspected swine flu or influenza, body temperature ≥38 °C, known sensibility to one of the ingredients of the study product, pregnancy or nursing, use of immunosuppressants or stimulants, use of pre- and probiotics.

The sample size calculation was determined by the previously observed effect size and the requirements of a significance level of 5.0 % (two-sided) and a power of 80 % [5]. One study site missed the predetermined difference of the average cold incidence between study groups and, thus, was excluded from further analysis. From the remaining 164 subjects, two were excluded from the intention to treat (ITT) population due to absence of available values after baseline leaving 162 subjects for the ITT analysis. Furthermore, 16 subjects were excluded from the per protocol (PP) analysis, either due to early termination (*n* = 5) or due to poor compliance (*n* = 11), resulting in 146 subjects. Hence, 146 subjects remained for PP analysis.

The beta-glucan preparation is an insoluble (1,3)-(1,6)-beta-glucan made from brewers’ yeast (*S. cerevisiae*), with a purity of at least 85 % on dry matter (branching factor approximately: 1,3 [backbone]: 1,6 [side chain]: 1,3/1,6 [branching] = 10:1:0.6). Moreover, it contains <2 % α-d-Mannan, <3 % fat, <2 % protein and <2 % ash on dry matter. The dry matter is more than 94 %.

Subjects were randomly assigned to receive a total of 900 mg of either insoluble yeast beta-glucan (Yestimun^®^ provided by Leiber GmbH, Germany) or placebo, each provided in two capsules per day, which were consumed at breakfast and supper. Detection of clinical effects like a reduction in common cold infections was demonstrated recently at a daily dosage of 900 mg insoluble beta-glucan [5].

The placebo product was maltodextrin. Verum and placebo capsules were identical in shape, colour, size and taste. The randomization code was created by an independent statistician prior to study start. The random numbers in blocks of 4 were randomized in a 1:1 ratio to the two study groups.

The study was approved by the Ethic Committee of the Charité, Berlin, Germany and was carried out in accordance with the Helsinki declaration and ICH GCP E6. Participants gave written informed consent prior to the study. The study was registered in the International Standard Randomized Controlled Trial Number Register (http://www.isrctn.org/) as ISRCTN16094368.

### Study design

The study was a multi-centric, randomized, double-blind, placebo-controlled study with two parallel arms in healthy subjects with recurring common cold episodes. The study was performed between October 2010 and May 2011. The subjects were enrolled at 7 study sites. During the study period of 16 weeks, a total of 3 routine visits were performed: at baseline, after 8 weeks and at the end after 16 weeks. In addition, one episode visit was conducted on the 5th day of each cold episode. Hence, the total number of visits per subject varies depended on the number of common cold episodes. During an infection, the subjects were instructed to record and assess their cold symptoms for a period of 14 days for each occurring episode.

Compliance was determined by counting returned capsules. Sufficient compliance was considered if >75 and <125 % of the capsules were consumed.

### Outcome measures

The primary objective of the present study, the incidence of common cold episodes, was defined as the number of common cold infections during the study period. Further, severity and duration of cold episodes were assessed.

 A cold episode was defined by the occurrence of at least two of the following cold symptoms: sore throat, feeling of lump in the throat/difficulty swallowing, hoarseness, cough, rhinorrhea or nasal congestion. These symptoms had to be rated with at least one point. Upon occurrence of the cold episode, the subjects had to document ten predefined common cold symptoms (headache, joint pain, sore throat, feeling of lump in the throat/difficulty swallowing, hoarseness, cough, rhinorrhea, nasal congestion, cold-related sleeping difficulties, fever) in an episode diary. The evaluation was carried out on a rating scale (0 points = symptom free, 1 point = mild symptoms, 2 points = moderate symptoms, 3 points = severe symptoms). The occurrence of fever was rated three points. By summation of the scores of the individual symptoms, a sum of scores (=total score) was calculated. All episodes were documented by the participants in a diary and confirmed by an investigator during the episode visit on the 5th day of each cold episode. The investigators examination includes the auscultation of the lungs, the control of the subject’s diary, the recording of possible vaccination against influenza/swine flu, the assessment and documentation of cold symptoms, any use of antibiotics as well as any changes in the concomitant illness and medications and the recording of sick leave days.

In seven cases, a cold episode occurred within first 4 days of treatment. Due to the presumed date of infection prior to intake of the investigational product (prophylactic effect not assessable), these episodes were not considered in the analysis.

As concurrent variables, the efficacy and tolerability of the investigational product was evaluated by both the subjects and the investigators at the end of the study. The following ratings were used for it: “very good” “good”, “moderate” or “poor”. The safety and tolerability of the product was additionally evaluated by the documentation of adverse events.

### Statistical analysis

All the variables contained in the data collection were presented descriptively using their statistical key data or their frequency distribution and statistically analysed in view of the group specific differences (*p*_χ_-value). The Mann–Whitney *U* test was employed to test for between-groups comparison (*p*_u_). All statistical analyses were carried out on both the ITT and PP collective. Statistical analyses were performed with SPSS (SPSS for windows, Release 19, LEAD Technologies Inc). Values of *p* < 0.05 were considered significant.

## Results

### Characteristics of the study participants at baseline

Of the 162 subjects analysed, 50 were men and 112 were women. All subjects reported that they had experienced at least three cold episodes in the 6 months prior to beginning of the study. The age, BMI and mean waist circumference values did not differ between study arms at baseline (Table 1).

**Table 1:** Mean (± SD) baseline characteristics of the study population

	All subjects (*n* = 162)	Verum (*n* = 81)	Placebo (*n* = 81)	*p*_u_
Sex (m/f)	50/112	23/58	27/54	0.496
Age (year)	43.2 ± 15.7	43.7 ± 15.1	42.7 ± 16.3	0.696
BMI (kg/m^2^)	25.2 ± 5.1	25.3 ± 5.4	25.1 ± 4.9	0.993

### Incidence of common cold infection

**Fig. 1:**
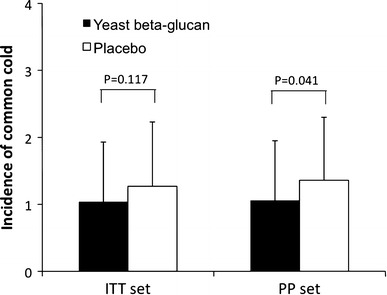
Number of common cold infections during a 16-week yeast beta-glucan or placebo supplementation according to the ITT and the PP set, values are mean ± SD

The intake of yeast beta-glucan significantly reduced the number of common cold episodes (1.06 ± 0.89) as compared to placebo (1.36 ± 0.94) in the PP population (*p* = 0.041) (Fig. 1). Regarding the ITT collective, the number of episodes was reduced by 19 % in the beta-glucan group (1.04 ± 0.89) as compared to the placebo group (1.27 ± 0.96; *p* = 0.117).

### Severity and duration of common cold episodes

A slight improvement in the mean total symptom score in the active (7.25 ± 4.39) compared to the placebo group (8.56 ± 5.31) over the entire study in the ITT population was prominent, which was, however, statistically not significant (*p* = 0.125). Cold-related sleep difficulties were significantly reduced in the beta-glucan group (*p* = 0.028) (Table 2).

**Table 2:** Mean (± SD) symptom scores during the entire study period (ITT)

Cold symptoms	All subjects mean ± SD	Verum mean ± SD	Placebo mean ± SD	*p* ^a^
Headache	0.83 ± 0.84	0.79 ± 0.82	0.86 ± 0.85	0.569
Joint pain	0.63 ± 0.82	0.55 ± 0.77	0.70 ± 0.86	0.236
Sore throat	0.96 ± 0.90	0.93 ± 0.90	0.99 ± 0.90	0.591
Feeling of lump in the throat	0.72 ± 0.91	0.69 ± 0.91	0.75 ± 0.92	0.594
Hoarseness	0.59 ± 0.79	0.51 ± 0.72	0.65 ± 0.84	0.315
Cough	1.03 ± 1.02	0.93 ± 1.00	1.12 ± 1.04	0.215
Rhinorrhea	1.20 ± 1.04	1.08 ± 1.06	1.29 ± 1.02	0.147
Nasal congestion	1.11 ± 0.97	1.07 ± 0.98	1.15 ± 0.96	0.559
Cold-related sleeping difficulties	0.92 ± 0.97	0.75 ± 0.92	1.06 ± 0.99	0.028
Total	7.97 ± 4.95	7.25 ± 4.39	8.56 ± 5.31	0.125

^a^ *p* values (Mann–Whitney *U* test) for differences between study arms

The superiority of beta-glucan supplementation was also confirmed in the subgroup analyses. There were significantly fewer subjects with at least one severe episode (episode severity >10 points in the total symptom score) in the active group (17.6 %) as compared to the placebo group (31.1 %) (*p* = 0.028). The duration of an episode did not differ between the active group and placebo (8.8 ± 2.9 vs. 9.2 ± 2.9 days, respectively; *p* = 0.470).

### Global evaluation of the efficacy during episodes (ITT)

At the end of the study, the global assessment of efficacy for yeast beta-glucan supplementation was rated as “very good” or as “good” by 83.7 % of participants and by the investigators for 76.1 % of the subjects. For placebo, the efficacy was rated as “very good” or “good” by 63.5 % subjects in self-assessment and by the investigators for 64.1 % of the subjects. Both physicians and participants rated the efficacy of yeast beta-glucan better than the placebo (*p*_χ_ = 0.004 and *p*_χ_ = 0.012, respectively).

### Safety evaluation

All measured clinical parameters, body weight, temperature, heart rate and blood pressure remained almost constant during the study, with no significant differences between the two study populations (data not shown).

The global assessment of tolerability for both treatments was rated at the end of the study. Both investigators and subjects rated the tolerability of the study products as equal (*p*_χ_ = 0.552 and *p*_χ_ = 0.487, respectively).

A total of 31 adverse events documented in 28 subjects occurred during the study period. Seventeen of these occurred in the active group and fourteen in the placebo group. Three adverse events in the verum group (rash on both arms and shoulders, gastric pressure after taking study product, minimal allergic rash on the left hand) and two in the placebo group (mucus in the nose since the intake of the study product, dry throat) were classified as probably related to the intake of the investigational product. The two study groups did not differ in the proportion of subjects with adverse events (*p*_χ_ = 0.626).

## Discussion

The present placebo-controlled, randomized, double-blind intervention study provides clinical evidence that supplementation with yeast (1,3)-(1,6)-beta-glucan helps to reduce the occurrence of symptomatic common cold infections by 25 % as compared to placebo (Fig. 1). In addition, consumption of yeast (1,3)-(1,6)-beta-glucan caused a milder progression of the severe common cold episodes. This is in line with a recently published trial demonstrating the prophylactic effect of the same yeast (1,3)-(1,6)-beta-glucan preparation on the incidence and severity of common cold infections [5]. Thus, since the susceptibility to get a common cold is closely related to the body’s immune status [6, 7], both studies independently suggest the potential of yeast (1,3)-(1,6)-beta-glucan to stimulate the host immune system in order to provide defence against common cold viral attacks.

The innate immune response to invading pathogens depends largely on the recognition of compounds commonly known as pathogen-associated molecular patterns (PAMPs) by so-called pattern recognition receptors (PRR) [8]. Beta-glucans are considered as one of the major PAMPs for the PPR-mediated sensing of fungal infection. One of the key PRR for beta-glucans are suggested to be dectin-1 [9, 10], although other receptors like toll-like receptors (TLR) and complement receptor 3 (CR3) also play a major role [11, 12]. According to in vitro studies, binding of beta-glucan to dectin-1 activates antigen-presenting cells like macrophages and dendritic cells and induces a cascade of innate and adaptive immune response which ultimately results in the eradication of the pathogen [10, 13]. Recently, Goodridge et al. demonstrated that although both soluble as well as particulate beta-glucan polymers are able to bind to the dectin receptor, only the particulate beta-glucan was able to activate the dectin-1 signalling, which in turn triggers phagocytosis [10].

The exact mode of action, however, depends at least in part on the route of administration. In terms of oral administration, the protection is primarily explained by the interaction of beta-glucans with pinocytic M-cells located in Payer’s patches of the small intestine. After activation, these cells are transported to lymph nodes where other cells of the immune system like macrophages are activated [14, 15]. In mice, orally administered yeast beta-glucan were taken up by gastrointestinal macrophages and shuttled to spleen, lymph nodes and bone marrow [16]. Within the bone marrow, the beta-glucan molecules were processed to small soluble biologically active fragments. This in turn is suggested to trigger the innate and adaptive immune response. Indeed, orally administered yeast beta-glucans induced the innate immunity as shown by a higher phagocytic activity and oxidative metabolism of immune cells in rats [17]. The positive effect of yeast beta-glucan administration on the specific humoral and cellular immunity was further demonstrated. In weaned piglets, yeast beta-glucan administration increased serum levels of antibodies and modulated immune response by mitigating the increase of pro-inflammatory cytokines after immunological challenge [18]. Fleischer and co-workers studied the effect of yeast beta-glucan on the immune system of chickens and pregnant pigs [21, 22]. As a result, administration with yeast beta-glucan increased the level of immune globulins and thereby reduced the infection rates and mortality in growing chicks, breeding pigs and their offspring.

A proprietary insoluble yeast beta-glucan supplementation reduced the symptoms associated with upper respiratory tract infections but not the incidence in marathon runners [19]. Likewise, the same preparation did not impact the incidence of common cold in healthy subjects in two independent studies [20, 21]. Even though the number of infections was not different, none of the subjects supplemented with beta-glucan missed school or work due to colds as opposed to placebo in the study conducted by Feldman et al. [21]. This is in contrast to our study, demonstrating an effect size of 25 % with yeast beta-glucan supplementation. The difference might be explained by varying study conditions, study populations, applied dosages and the purity of the applied beta-glucan preparation. Indeed, the immunomodulatory activity of beta-glucans from the same source might differ considerably by the level of purity [22].

Overall, the present study proved the safety and tolerability of an insoluble (1,3)-(1,6)-beta-glucan preparation made of brewers’ yeast. This is in line with results of controlled animal and human studies demonstrating no signs of toxicity after oral application of yeast (1,3)-(1,6)-beta-glucan preparation [5, 17, 19].

In conclusion, our study in healthy subjects, together with a very recently published trial [5], confirms the postulation of an immunostimulatory effect of a highly purified yeast (1,3)-(1,6)-beta-glucan preparation. This indicates the improvement in the body’s defence against pathogens. Notably, a reduction in common cold episodes by 20–25 % was independently demonstrated for two independent study populations in two different winter seasons, which underlines the robustness of the effect and biological relevance of this yeast (1,3)-(1,6)-beta-glucan preparation.
